# Retrospective Study of Tick Bites Associated with Neurological Disease in a Level Three University Hospital in Switzerland

**DOI:** 10.3390/idr15020016

**Published:** 2023-03-01

**Authors:** Patrick Thalmann, Simone Ehrhard, Artur Summerfield, Meret Elisabeth Ricklin

**Affiliations:** 1 Department of Emergency Medicine, Inselspital, Bern University Hospital, University of Bern, 3010 Bern, Switzerland; 2 Department of Immunology, Institute of Virology and Immunology, 3147 Mittelhäusern, Switzerland; 3 Department of Infectious Diseases and Pathobiology, Vetsuisse Faculty, University of Bern, 3001 Bern, Switzerland; 4 Multidisciplinary Center for Infectious Diseases (MCID), University of Bern, Hallerstrasse 6, 3012 Bern, Switzerland

**Keywords:** emergency department, tickborne disease, neurological symptoms, Borreliosis, tick borne encephalitis, public health

## Abstract

Background: Ticks represent very important vectors of human and zoonotic pathogens, and tick-borne diseases (TBDs) are diagnosed with increasing frequency. Therefore, the aim of this retrospective study was to describe patients presenting with a complaint of tick bite in the emergency department (ED) of a large university hospital in Switzerland. Methods: Data were collected by searching for keywords in the routine clinic database to identify cases from 1 July 2012 to 30 June 2020. The patients’ data were screened for preexisting diseases and demographic and clinical characteristics. Results: We included 415 patients collected over a period of 8 years, with highest admission frequencies from May to July. Of these, 82% were outpatients, 15.9% admitted to a hospital ward, and five to intensive care. The patients were allocated to three groups. The first group represented patients with erythema chronica migrans (ECM), which is pathognomonic for Lyme borreliosis (n = 45). Accordingly, 89% of cases in this group of patients were treated with antibiotics. The second group represented patients with other tick bite-associated erythema (n = 139). In this group, no particular clinical symptoms or laboratory findings were found. Finally, the largest group represented patients in which the tick bite was no longer visible (n = 201). This group of patients had significantly more evidence of neurological disorders (52%) and were treated at a higher rate with non-steroidal anti-inflammatory (29%) or antiviral (13%) drugs. Although the vaccination status for tick-borne encephalitis virus (TBEV) was not systematically evaluated, at least 10% of the latter group was vaccinated, indicating another source for neurological disease. Furthermore, only 14% of the tested patients were positive for IgM or IgG against TBEV. Conclusion: This retrospective study indicates the presence of many undiagnosed neurological diseases following tick bites that could be caused by TBEV or an unknown infectious agent. Taken together, although tick bites were not very frequently seen in the present tertiary ED, the frequent presence of neurological symptoms demands a more systematic assessment of vaccination status and TBEV serology as well as further diagnostic evaluations in patients that report tick bites and neurological symptoms.

## 1. Background

Next to mosquitos, ticks are the most important arthropod vectors of human and zoonotic pathogens globally [1]. Among all human-biting tick species, *Ixodes ricinus* is the most common species in Europe. Next to *Ixodes ricinus*, *Rhipicephalus sanguineus*, *Hyalomma marginatum*, and *Dermacentor reticulatus* are frequent species in Europe [2].

Ticks can be the vector of many important tick-borne pathogens (TBPs) that cause several well-known infectious diseases, generally referred to as tick-borne diseases (TBDs). The transmitted pathogens depend on the tick species. The most relevant TBPs in Switzerland are *Borrelia spp.*, causing Lyme borreliosis (LB), tick-borne encephalitis virus, causing tick-borne encephalitis (TBE), *Coxiella burnetti*, causing Q-fever, and *Francisella tularensis*, causing tularemia. All of these TBPs are transmitted by *Ixodes ricinus* [3]. *Dermacentor reticulatus* may transmit *Babesia canis*, leading to babesiosis [4]. *Hyalomma marginatum* can transmit *Rickettsia aeschlimannii*, which has increasing prevalence and can cause liver dysfunction in humans [5].

According to the Swiss Federal Office of Public Health, around 13,000 people were diagnosed with LB, 439 with TBE, 59 with Q-fever, and 45 with tularemia in 2020 [6]. No such data are available concerning other TBDs. Clearly, tick bites are steadily increasing in Switzerland, and TBDs are increasing globally, including in Europe [7]. The emergence of more pathogen transmission of the species *Borrelia*, *Rickettsia*, *Ehrlichia*, and *Anaplasma,* as well as many tick-borne viruses, is likely [8]. An example of the latter is the recent discovery of Alongshan virus in *Ixodes ricinus* ticks in Switzerland [9].

Of all TBDs, LB is the most frequent in Switzerland. It can affect various organs and result in dermatological, musculoskeletal, neurologic, and cardiac involvement [10]. If untreated, LB has an increased risk of long-term morbidity. Antibiotics are the treatment of choice, however early manifestations of LB may heal spontaneously [1]. TBE results in unspecific symptoms such as fever, headache, and gastrointestinal symptoms [11]. Tularemia often results in regional lymphadenopathy. Fatigue, sudden onset of chills, fever, headache, and vomiting are possible systemic symptoms. Antibiotics are the treatment of choice [12]. An infection with Q-fever remains mostly asymptomatic but can manifest as a febrile illness, pneumonia, hepatitis, or endocarditis [13]. Diagnosis of these diseases is based mainly on serology [11,13,14].

Tick bites are usually not painful, and just below 50% of patients with TBD can recall being bitten [15,16]. Furthermore, symptoms of TBD can be unspecific. For these reasons, TBDs can often be misdiagnosed [17]. Consequently, with the aim of obtaining information around disease frequency and associated clinical and laboratory findings and to identify diagnostic gaps, the present retrospective study collected and described all TBD cases in a Swiss university hospital emergency department (ED) over a period of 8 years. We include all patients presenting with a tick bite noticed in the four weeks prior to presentation and describe the symptoms, laboratory parameters, diagnostic procedures, hospitalization, treatment, and clinical outcomes.

## 2. Methods

**Figure 1:**
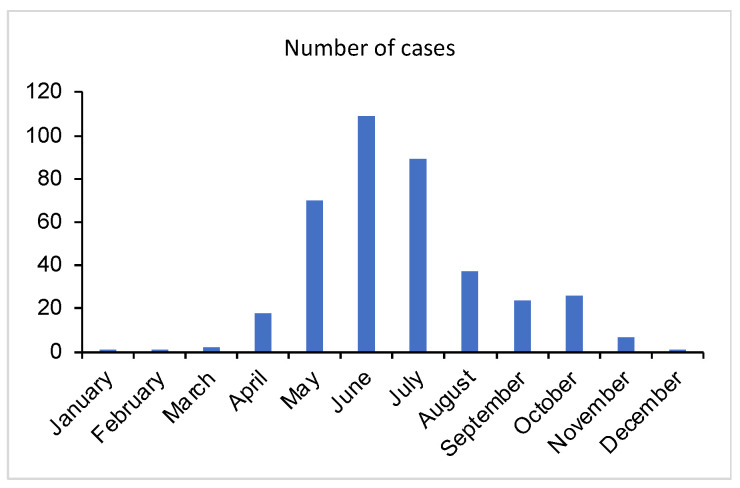
Histogram of cases by admission date. Of the 4698 total, 385 patients were selected based on the case definition relating to potential TBDs.

This retrospective study was performed at a level three university hospital ED in Bern, the capital of Switzerland. Data were collected by searching the routine clinic database for the following keywords: “Zecken”, “Zeckenbisse”, “Zeckenstiche”, “Erythma migrans”, “Borrelio*”, “Tularämie”, “Anaplasm*”. The search was executed by the Insel Data Coordination Lab (IDCL), identifying cases from 1 July 2012 to 30 June 2020. Patients were included if a tick bite was recorded in the four weeks prior to consultation. The patients’ data were screened for preexisting diseases (defined as diseases that were under medical treatment at the date of assessment) and demographic characteristics, such as month of admission, sex, age group, hospital admission, TBE vaccination status, and further consilia of other specialties. Patients with missing informed consent were excluded. Of the 4698 total, 385 patients were selected based on the above case definition relating to potential TBDs. The distribution of the cases by month is shown in Figure 1.

We grouped these patients as follows: (1) Patients presenting with erythema chronica migrans (ECM), typical for Borreliosis (group “ECM”; 45 patients), (2) patients with other tick bite-associated erythema (group “erythema”; 136 patients), and (3) patients in which no skin lesions typical of tick bites were found (group “none”; 201 patients).

Reports were checked for the main symptoms, such as nausea, vomiting, diarrhea, pain in different locations (headache, abdominal pain, thoracic pain, other pain locations), pruritus, dyspnea, arthralgia/myalgia, symptoms of cold, exanthemas (erythema at bite site, ECM, other exanthema), sensitivity disorders, neurological signs (vertigo, psychiatric problems, double vision), fatigue, and facials paralysis. Furthermore, clinical signs such as bradycardia (pulse < 60/min), tachycardia (pulse > 90/min), high blood pressure (>140 mmHg systolic and >90 mmHg diastolic), fever (T > 38 °C), meningism, and GCS < 15 were assessed. The patient reports were also evaluated for other diagnostic procedures, such as imaging (computed tomography (CT), ultrasound (US), magnetic resonance tomography (MRT), and radiography), lumbar puncture (LP), and electrocardiography (ECG).

If available, different laboratory parameters were included (leukocytes [3–10.5 G/L], proteinuria [neg], C-reactive protein (CRP) [<5 mg/L]). The reference values of the Inselspital Bern were used.

TBEV serology tests were considered as positive if IgM, IgG, or both were positive. Due to the low number of positive sera, subdivision into acute (only IgM positive) and chronic (IgG positive) infections was not performed. No data on isotype switching from IgM to IgG was available as follow-up data for seroconversion was lacking. ECM was defined as an erythematous skin lesion around the tick bite, which develops days to weeks after exposure [1]. Indications for neurological problems were taken together in a new category, “evidence of neurological disorder”. These included neurology consultation, sensitivity disorders, and meningism in combination with headache, facial paralysis, GCS < 15 and diagnoses in the hospital report classified as “neurological”.

Finally, medications prescribed by the ED were grouped into categories of non-steroidal anti-inflammatory drugs (NSAIDs), corticosteroids, opioids, antibiotics, antiparasitics, virostatics, and steroids. Follow-up data, if available, were assessed.

For multiple comparisons, a chi-squared test was performed using STATA 14.2 (StataCorp, College Station, TX, USA).

The study was performed according to Swiss law and approved by the local ethics commission (Kantonale Ethikkommission Bern Nr: 2020-01668, Bern, Switzerland).

## 3. Results

### 3.1. Number of Cases by Month of ED Admission

The number of cases per month is shown in Figure 1. Most admissions were registered in the months of May to July and peaking in June, which represents the tick bite season.

### 3.2. Patient Characteristics

Of the 385 patients included, males were slightly overrepresented at 53.26%. The most frequently represented age group was 26–35 years old (Table 1). The average stay in the ED was 3–4 h with only a few staying more than 8 h. Furthermore, most of the patients were treated as outpatients with only 16% being transferred to a hospital ward and even fewer (1.21%) being transferred to an intensive care unit. Thirty-three patients (8.05%) had a history of recorded TBE vaccination. If further consultation had been requested, neurology services were the most frequently used. Demographic details of the patient population are presented in Table 1.

### 3.3. Main Symptoms in Patients Classified by Tick Bite-Associated Skin Lesions

When grouping the patients by skin lesions, we identified 45 patients with ECM (group “ECM”), 135 patients with other tick bite-associated erythema (group “erythema”; 136 patients), and 201 patients (52.08%) in whom no skin lesions typical of tick bites were found (group “none”).

For patients in the group “erythema”, we found no increased association with any of the available parameters (Table 2). This also applied to “ECM” patients, with the exception of a high percentage of antibiotic therapy. Importantly, fewer patients without skin lesions, in the group “none”, were classified as “infectious disease”, but with approximately 50% clearly more recorded as neurological in nature. The higher number of inpatients and levels of treatment with NSAIDs and antivirals underlines that disease status was considered relatively severe in some patients. The data would therefore indicate a possible symptomatic infection with TBEV in some of these patients, although only 14% of those tested were serologically positive. This, and the observation that 10% of the tested patients were TBEV vaccinated, point to a possible alternative etiology of the observed neurological disease. However, both the TBEV serology and vaccinology data are quite incomplete, making deeper analysis of the associations impossible.

Taking these results together, we have identified a considerable number of undiagnosed neurological diseases following tick bites that could be caused by TBEV or an unknown infectious agent possibly transmitted by tick bites.

## 4. Discussion

The present retrospective study was performed in an ED of a Swiss level three university hospital which treats about 45,000 patients per year [18]. On average, 50 patients visited this ED per year due to a tick bite in the four weeks prior to presentation. The seasonal distribution of these cases was in line with the presence of ticks in the environment. The fact that the main age group affected was 26–45 year-old adults can possibly be explained by this age group spending more time in nature. Over the period of 8 years, TBDs resulted in five intensive care patients. Of note, a clear causality between tick bite and ICU hospitalization was not ascertainable. Although this data indicate that TBDs have been a minor health problem in Switzerland, the predicted climate change and increased human population are expected to make TBDs a growing health problem [19,20].

Based on the observation that 45 patients had ECM, which is pathognomonic for LB, it could be estimated that around 10% of all our patients with tick bites had LB. In the Netherlands, Hofhuis et al. estimated the risk of infection with *B. burgdorferi* after a tick bite to be 5.1% [21], and Wilhelmsson et al. confirmed these results by identifying borrelia infection in five percent of tick bite patients [22]. It is important to note that our data do not permit an estimation of the frequency of LB following tick bites in the catchment area of the hospital. Therefore, further investigations are required to determine the risk of LB in Switzerland. In this context, it must be noted that seropositivity does not allow confirmation of active Lyme borreliosis [23].

Interestingly, we noted no significant association between any symptom or laboratory finding with the group of patients reporting non-ECM erythema, and this was in contrast to patients reporting a tick bite without erythema. It was striking to observe that over 50% of this largest group of patients showed evidence of neurological symptoms/disease. Although the estimated time point of the tick bites was not recorded, it is possible that these patients were infected earlier compared to those with erythema, hence showing some symptoms, or infected by an undetected subtype of the TBE virus [24]. The incubation time of TBE has been reported to be 4–28 days, mostly between 7 and 14 days [25]. Further studies are warranted to clarify this point as well as the etiology of symptoms.

The ECM group of patients were not associated with a higher rate of hospitalizations and had a lower but still considerable frequency (20%) of neurological complications. An example of such a manifestation that can be associated with Lyme borreliosis is acute transverse myelitis [26]. Future prospective studies are required to address the nature and frequencies of such complications.

Headache and neurological signs were both significantly more frequent in hospitalized patients, indicating that these symptoms are potential markers for a more severe course of disease. However, although headache has been identified as a symptom of almost all TBD in many studies, the connection to a severe course has, to our knowledge, not been clarified yet [1,27,28,29,30]. Our findings could indicate that headache, although very unspecific, could be an early sign of a potentially severe case. However, headache is a broad symptom and further investigation regarding periodicity, exact location, and co-symptoms such as phono-/photophobia, vertigo, etc. needs to be performed.

Altogether, the relatively higher frequency of neurological diagnoses that required treatment in the group of patients without erythema demonstrates the presence of undiagnosed neurological infections. Due to the considerable data gaps in serology and vaccination status, we cannot estimate how many of these cases represent TBEV infections. Again, this requires prospective studies to be designed to address this question. For these studies, systematically assessing TBEV vaccination will be important as this vaccine is reported to have an efficacy of over 91% in Switzerland [31]. Nevertheless, positive TBE serology was more frequent for inpatients than outpatients (69.5% and 39.3%, respectively), possibly indicating that some seropositive patients had ongoing TBE. In rare cases reported in Australia, Canada, the United States, and other parts of the world, neurological symptoms such as paralysis can also be the result of tick toxicosis by certain venomous species [8]. Although this should be taken into consideration as a differential diagnosis, symptoms usually disappear 24 h after removal of the tick(s) [8,32].

A main limitation of our study is the incompleteness of the datasets, which limits the possibility of drawing conclusions with respect to infectious agents. A systematic assessment of the serological and vaccination status as well as possible coinfections represent an important information gap. This is often seen in retrospective studies. Furthermore, although our study was performed at one of the largest emergency departments in Switzerland, the sample size was relatively low, making further in-depth statistical analyses not possible.

## 5. Conclusions

In conclusion, the data indicate a considerable number of undiagnosed neurological diseases following tick bites that could be caused by TBEV or an unknown infectious agent transmitted by tick bites. Our data demand a more systematic diagnostic evaluation of such cases. In particular, a systematic assessment of TBEV vaccinations as well as serology status is warranted in patients reporting a tick bite in association with signs of neurological disease. If the presence of unidentified infectious agents is consolidated, we recommend further diagnostic evaluations in patients that report tick bites. This is also important because the incidence of TBDs is expected to continue to rise in the future due to climate change and through imported cases [33]. Certainly, improving the collaboration between human and veterinary medicine is also advisable since a rise of TBDs in one sector is connected to a rise in the other [34]. This will enable a more efficient tick surveillance system and TBP screening. Such data will help to define and improve prevention and treatment strategies for TBDs.

## Figures and Tables

**Table 1:** Demographic data and patient characteristics.

Sex	
Female	180 (46.75)
Male	205 (53.24)
Age groups (years)	
16–25	54 (14.02)
26–35	92 (23.89)
36–45	76 (19.74)
46–55	65 (16.88)
56–65	45 (11.69)
66–75	33 (8.57)
≥75	20 (5.19)
Hospital admission	
Outpatient	321 (83.37)
Hospital ward	64 (16.6)
Intensive care	5 (1.29)
TBE vaccination status	
Vaccinated	32 (8.31)
Non vaccinated	353 (91.69)
Consilia of specialties (can be multiple)	
None	320 (83.11)
Neurology	22 (5.81)
Infectious diseases	21 (5.08)
Others *	24 (5.81)

* Others: ENT: ear nose throat, surgery, dermatology, psychiatry, cardiology, gynecology, rheumatology, gastro-enterology, oncology, hematology.

**Table 2:** Main clinical symptoms by patient group shown as absolute and percentage values relative to the group (in parenthesis).

Symptom	Group “Erythema”	Group “ECM”	Group “None”
Infectious disease diagnosis	115 (82.7)	38 (84.4)	88 (43.8) *
Evidence of neurological disorder	29 (20.9)	9 (20.0)	104 (51.74) **
Facial paresis	1 (0.7)	0 (0)	7 (3.5)
Sensation disorder	4 (2.9)	2 (4.4)	31 (15.4) **
Fever	4 (2.9)	2 (4.4)	21 (10.5)
Headache	24 (17.3)	8 (17.8)	81 (40.3) **
Meningism	2 (1.4)	0 (0)	12 (6.0) *
Pruritus	18 (13.0)	8 (17.8)	7 (3.5) *
TBEV positive serology	5 (3.6)	0 (0)	28 (13.9)
TBEV vaccine	10 (7.2)	2 (4.4)	20 (10.0)
Lumbar puncture	4 (2.9)	4 (8.9)	30 (14.9) *
MRI	10 (7.2)	2 (4.4)	50 (24.9) *
Normal imaging	3 (2.15)	3 (6.7)	44 (21.9) **
Leukocytosis	6 (4.3)	0 (0)	32 (15.9)
Elevated CRP	20 (14.4)	8 (17.8)	67 (33.3)
Proteinuria	1 (0.7)	1 (2.2)	13 (6.5)
GI-Symptoms	17 (12.2)	8 (17.8)	62 (30.8)
Inpatients	8 (5.8)	3 (6.7)	53 (26.4) **
NASID treatment	16 (11.5)	5 (11.1)	58 (28.9) **
Corticosteroids	0 (0)	0 (0)	1 (0.5%)
Antiviral treatment	3 (2.2)	0 (0)	27 (13.4) **
Antibiotics	32 (23.0)	40 (88.9) **	42 (20.9)

* *p* ≤ 0.05; ** *p* ≤ 0.01.

## Data Availability

Not applicable.

## List of Abbreviations

TBP	tick-borne pathogen
TBD	tick-borne disease
LB	Lime borreliosis
TBE	tick-borne encephalitis
ED	emergency department
IDCL	Insel Data Coordination Lab
ECM	erythema chronica migrans
CT	computed tomography
US	ultrasound
MRT	magnetic resonance tomography
LP	lumbar puncture
ECG	electrocardiography
CRP	C-reactive protein
NSAID	non-steroidal anti-inflammatory drug
ENT	ear, nose, and throat
